# The effect of the COVID-19 pandemic on primary care physicians in Israel, with comparison to an international cohort: a cross-sectional study

**DOI:** 10.1186/s13584-022-00543-8

**Published:** 2022-09-20

**Authors:** Limor Adler, Shlomo Vinker, Anthony D. Heymann, Esther Van Poel, Sara Willems, Galia Zacay

**Affiliations:** 1 Department of Family Medicine, Sackler Faculty of Medicine, Tal Aviv University, Tel Aviv, Israel; 2 Department of Family Medicine, Maccabi Healthcare Services, Tel Aviv, Israel; 3 Headquarters, Medical Division, Leumit Healthcare Services, Tel Aviv, Israel; 4 Department of Family Medicine, Meuhedet Healthcare Maintenance Organization, Tel Aviv, Israel; 5 Department of Public Health and Primary Care, Ghent University, Ghent, Belgium; 6 Quality and Safety Ghent, Department of Public Health and Primary Care, Ghent University, Ghent, Belgium

**Keywords:** Primary care physicians, Community clinics, COVID-19, Physicians’ well-being, Telemedicine, PRICOV-19

## Abstract

**Background:**

The COVID-19 pandemic had a major impact on primary care and primary care physicians (PCPs) in Israel and around the world. There is paucity of information regarding treatment of patients with COVID-19 in the community, since most research was performed in hospitals. The aim of this study was to describe the Israeli PCPs’ experience.

**Methods:**

This study is a part of an international cross-sectional study, the PRICOV-19. A translated version of the questionnaire was distributed among Israeli PCPs from December 2020 to July 2021. In this study, we describe the Israeli results and compare them to the international results.

**Results:**

5,961 respondents from 29 countries answered the questionnaire, 94 from Israel, with an Israeli response rate of 16%. Israeli PCPs reported an increase in use of telemedicine from 11 to 49% during the COVID epidemic. PCPs also reported a decline in their wellbeing; absence of secured time slots for keeping updated; perception that the Ministry of Health guidelines were a threat to the staff wellbeing and organization of practice and delays in the examination of non-COVID urgent cases.

**Conclusions:**

The findings of this study raise concerns regarding the PCPs experience and may form the basis for an improved process of care. Guidelines for proper usage of telemedicine, substitutes for the physical examination and procedures for minimizing delayed patient examination for urgent conditions should be developed. Government directives and clinical guidelines should be communicated in a timely manner, with secured timeslots for physicians’ self-learning or updating. Ensuring physicians’ well-being in general should be an organization priority.

**Supplementary Information:**

The online version contains supplementary material available at 10.1186/s13584-022-00543-8.

## Background

On March 2020, the Israeli Ministry of Health (MoH) instructed the four healthcare maintenance organizations (HMOs) in Israel to continue delivering urgent and regular healthcare services with reduced physical contact and to prioritize the use of remote visits using modalities like telephone, video visits and offline requests using the HMOs Information Technology (IT) infrastructure. Such consultations were reimbursed like ordinary visits ensuring that the process would be supported by physicians. During the first lockdown, office-based care (face to face visits) volume was reduced by 50% [1]. Consequently, remote care, mainly carried out by phone, video, or electronic offline requests, increased substantially.

In Israel, most patients with SARS-CoV-2 infection or symptomatic COVID-19 were treated in the community, either by centralized remote COVID-19 managed care programs (by physicians and nurses) or by the patient’s personal primary care physician (PCP) (each physician treating his/her own patients) [2]. The same process happened in primary care throughout nations [3–5]. While treating patients with COVID-19 infection in addition to their ordinary daily tasks, PCPs faced challenges, including (a) Changes in practice management including an initial shortage of self-protection equipment and shortage of medical personnel; (b) Concerns regarding personal health risks from the virus and the risk of spreading the virus to their families, as well as an increased emotional burden; and (c) Concerns regarding the patient-centered approach in face of the increased volume of telemedicine [6, 7]. Another obstacle was paucity of information regarding treatment of patients with COVID-19 in the community, as most of the research was performed in hospitals, on hospitalized patients [8].

The COVID-19 pandemic had a psychological impact on health care providers, with increased depressive symptoms, COVID-related stress, tiredness, general anxiety, and lower levels of proactive coping [9–11]. It was noted that frontline healthcare providers who diagnosed, treated, and cared for patients with COVID-19 reported higher levels of mental health symptoms, including depression, anxiety, insomnia, and distress [11–13].

The aims of this study were to describe several aspects of the experience of PCPs in Israel during the pandemic; adaptations that were made, information flow, the impact of health ministry guidelines, physician’s well-being, and collateral damage such as delayed diagnosis and treatment.

## Methods

### Setting and study design

This study was performed as part of an international study, named PRICOV-19. PRICOV-19 was a multi-country cross-sectional study, which studied the response and adaptation of primary care practice organizations to the COVID-19 pandemic. The PRICOV-19 study used a convenient sample of physicians, and examined how practices were adapted to provide safe, effective, patient-centered, and equitable care. In addition, PRICOV-19 examined the pandemic's impact on the role and well-being of care providers [14].

From December 2020 to July 2021, we sent a link to an online questionnaire to PCPs through e-mails and WhatsApp messages. The physicians, who received the questionnaire, were members of a google group and a WhatsApp group. Both groups were used by PCPs for medical consultations among peers; these groups` members were mainly family-medicine specialists or residents, many of them were members of both groups. During June-July 2021, we also distributed a hard copy questionnaire during two national meetings of PCPs: an annual meeting of family medicine tutors and a clinical updates conference meeting. The participants of the groups and the meetings were working for all four HMOs in Israel. We sent the questionnaire to 600 PCPs. All data were collected and managed using REDCap (Research Electronic Data Capture) tools hosted at Ghent University [15].

### The questionnaire

The PRICOV-19 questionnaire was piloted among 159 practices in Flanders (Belgium). It consisted of 53 items arranged across six topics: (1) Infection prevention; (2) Patient flow for COVID and non-COVID care (scheduling appointments, triage, phone-calls, video visits, roles of different staff members, etc.); (3) Distribution of new information and protocols; (4) Communication with patients; (5) Collaboration; (6) Well-being of the respondent. We also collected characteristics of the respondent and the practice.

The well-being section was based on the Mayo clinic well-being index (an additional file contains this index [see Additional file 1]), which is a validated tool for assessing physicians’ well-being as well as identifying an increased risk for burnout [16–18]. The score ranges from -2 to 9, with higher scores indicating a higher degree of distress. An “at-risk score” is an index score of two or higher, which indicates higher risk for burnout, severe fatigue, suicidal ideation, and poor overall quality of life. We calculated the participants’ well-being score at the time they answered the questionnaire, and also asked them to answer the same questions for the pre-pandemic period.

The Ghent University team composed the questionnaire in English, and each local team translated the questionnaire to their language. LA (the Israeli principal investigator) translated the questionnaire to Hebrew, and SV (second author) reviewed and improved the translated questionnaire. Minor adjustments were made to fit the Israeli PCP population and healthcare system.

Participation in this study was voluntary and anonymous. Consent to participate was given at the beginning of the questionnaire with a full explanation given to participants. The original English version is available in an additional file (see Additional file 2).

### Statistical analysis

We report the sociodemographic characteristics of PCPs, as well as their responses using absolute numbers and percentages. All percentages in the results were calculated based on the valid answers with missing values excluded. We used the chi-square test to examine the associations between categorical variables and the Mann–Whitney test for variables that were not normally distributed. Spearman correlation test was used to estimate the correlation between nonparametric variables. A two-sided *p*-value smaller than 0.05 was considered statistically significant. The statistical analysis was performed with R version 4.1.0 (R Foundation for Statistical Computing).

### Ethics approval and consent to participate

The Research Ethics Committee of Ghent University Hospital approved the study protocol under the number BC-07617. The Israeli collection of data was approved by the ethical committee of Tel Aviv University. All methods were carried out in accordance with the ethical standards as laid down in the 1964 Declaration of Helsinki and its later amendments or comparable ethical standards. Consent to participate was granted by submission of a completed questionnaire.

## Results

We received 122 replies. Of these, we removed from the analysis 28 questionnaires that were almost completely empty and were left with 94 responses, a response rate of 16%. Altogether, 5,961 respondents from 29 countries completed the questionnaire with a response rate of 27.8%. Each country decided how to select its study participants, some addressed conference participants, while other sent the questionnaire to all or part of the country`s PCPs.

Tables 1, 2, 3, 4 and 5 present a comparison between the Israeli and international study populations and their responses to the questionnaire.

**Table 1:** Characteristics of Israeli and international respondents of the PRICOV-19 questionnaire

	Israeli respondents		Entire cohort	
	n	%	n	%
*Position in the practice*				
GP	47	50	3,309	55.5
GP trainee	18	19.1	242	4.1
Other	1	1.1	243	4.1
Missing	28	29.8	2,167	36.4
*Experience after residency (years)*				
0–4.99	21	22.3	525	8.8
5–9.99	7	7.4	438	7.3
10–14.99	5	5.3	441	7.4
15–19.99	10	10.6	440	7.4
20–24.99	2	2.1	526	8.8
25–29.99	8	8.5	386	6.5
30–34.99	6	6.4	402	6.7
35 or more	0	0	324	5.4
Missing	35	37.2	2,478	41.6
*Location of the practice*				
Big city	43	46.2	1,248	20.9
Suburbs	4	4.3	370	6.2
Small town	24	25.8	710	11.9
Mixed urban–rural	9	9.7	803	13.5
Rural	11	11.8	698	11.7
Missing	2	2.2	2,132	35.8
*Payment system*				
Fee for services	45	49.5	1,282	21.5
Capitation	39	42.9	1,570	26.3
Other	4	4.4	978	52.5
Missing	2	2.2	2,131	35.7
*Number of GPs in the practice*				
1–2	35	38	1,237	20.7
3–4	39	42.4	738	12.4
5–6	13	14.1	434	7.3
> 6	2	2.2	834	14
Missing	3	3.3	2,178	36.5
*Number of residents in the practice*				
0	22	23.9	1,921	32.2
1–2	56	60.8	1,184	19.9
> 2	3	3.3	447	7.5
Missing	11	12	2,409	40.4
*Number of paid staff*				
1–5	16	17.4	1,517	25.4
6–10	27	29.3	748	12.5
11–15	17	18.4	380	6.4
16–20	11	12	274	4.6
> 20	17	18.4	857	14.4
Missing	4	4.3	2,185	36.6

**Table 2:** Adaptations of primary care visits

	Israeli respondents		Entire cohort			*P* value
	n	%	n		%	
*To what extent this practice uses video consultations? Before the COVID-19 pandemic*						
Never	81	89	3,184		89.4	0.180
Less than once a week	3	3.3	244		6.8	
Weekly	3	3.3	88		2.5	
Daily	2	2.2	28		0.8	
Multiple times a day	2	2.2	19		0.5	
Total	91		3,563			
Missing	3		2,398			
*To what extent this practice uses video consultations? Since the COVID-19 pandemic*						
Never	46	51.1	2,151		60.6	0.092
Less than once a week	16	17.8	667		18.8	
Weekly	13	14.4	358		10.1	
Daily	8	8.9	249		7.0	
Multiple times a day	7	7.8	123		3.5	
Total	90		3,548			
Missing	4		2,413			
*When patients want to make an online appointment for this practice, they are shown a message informing them about which complaints they may (not) bring them to the practice*						
Yes	24	32.8	993		50.5	0.004
no	49	67.2	974		49.5	
Total	73		1,967			
Missing	21		3,994			
*Patients must state a reason when making an online appointment at the practice*						
Yes	6	7.3	1,256		60	< 0.001
no	76	92.7	836		40	
Total	82		2,092			
Missing	12		3,869			
*Patients must state a reason when making an appointment by phone*						
Yes	12	14.1	2,699			< 0.001
no	73	85.9	673			
Total	85		3372			
Missing	9		2,589			
*Patients who made an appointment and where it is unclear whether they pose a risk of infection are called beforehand to verify this*						
Never	35	42.2	416		12.8	< 0.001
Rarely	19	22.9	384		11.8	
Sometimes	13	15.7	460		14.1	
Usually	8	9.6	899		27.7	
Always	8	9.6	1,092		33.6	
Total	83		3,251			
Missing	11		2,710			
*Since the COVID-19 pandemic, my responsibilities in this practice increased*						
Strongly disagree	5	8.6	65		2.2	< 0.001
Disagree	7	12.1	129		4.3	
Neutral	11	19.0	441		14.8	
Agree	26	44.8	966		32.4	
Strongly agree	9	15.5	1,382		46.3	
Total	58		2,983			
Missing	36		2,978			
*Since the COVID-19 pandemic, I am happy with the task shifting in my professional role*						
Strongly disagree	4	7	314		10.7	0.104
Disagree	22	38.6	825		28.2	
Neutral	10	17.5	913		31.2	
Agree	17	29.8	650		22.2	
Strongly agree	4	7	222		7.6	
Total	57		2,924			
Missing	37		3,037			
*In this practice, one or more of the following initiatives were taken since the COVID-19 pandemic:*						
A list was compiled from the EMR (*) for at least one group of patients with a chronic disorder (e.g. all patients taking methotrexate and needing to be seen)						
Yes	38	54.3		927	28.6	< 0.001
No	32	45.7		2,318	71.4	
Total	70			3,245		
Missing	24			2,716		
*This practice contacted patients with a chronic condition who needed follow-up care*						
Yes	60	75		2,108	61.7	0.015
No	20	25		1,310	38.3	
Total	80			3,418		
Missing	14			2,543		
*This practice contacted psychologically vulnerable patients*						
Yes	22	33.3		1,114	33.9	0.92
No	44	66.7		2,170	66.1	
Total	66			3,284		
Missing	28			2,677		
*This practice contacted patients with previous problems of family violence or with a problematic child-rearing situation*						
Yes	6	10.2		482	15.8	0.242
No	53	89.8		2,577	84.2	
Total	59			3,059		
Missing	35			2,902		

The Israeli and the entire cohort responses to questions regarding adaptations of primary care visits during the pandemic, including telemedicine, increased responsibilities, shift in professional roles and initiatives taken during the pandemic

**Table 3:** Information and guidelines

	Israeli respondents		Entire cohort		*P* value
	n	%	n	%	
*In this practice, there is enough protected time is provided in the agenda(s) of GPs for reviewing new guidelines or going through relevant and reliable scientific literature. Before the COVID-19 pandemic*					
Strongly disagree	30	37.5	760	22.3	< 0.001
Disagree	31	38.7	882	25.9	
Neutral	6	7.5	675	19.8	
Agree	11	13.7	796	23.3	
Strongly agree	2	2.5	296	8.7	
Total	80		3,409		
Missing	14		2,552		
*In this practice, there is enough protected time is provided in the agenda(s) of GPs for reviewing new guidelines or going through relevant and reliable scientific literature. Since the COVID-19 pandemic*					
Strongly disagree	33	39.7	922	27.1	< 0.001
Disagree	31	37.3	836	24.6	
Neutral	4	4.8	533	15.7	
Agree	13	15.7	730	21.4	
Strongly agree	2	2.4	383	11.3	
Total	83		3,404		
Missing	11		2,557		
*Since the COVID-19 pandemic, how often is a meeting planned in this practice to discuss existing, new, or amended directives?*					
Never	26	31.3	439	13.0	< 0.001
Less than once a week	36	43.3	965	28.6	
Weekly	18	21.7	1,206	35.7	
Daily	3	3.6	678	20.1	
Multiple times a day	0		90	2.7	
Total	83		3,378		
Missing	11		2,583		
*The guidelines imposed by the government on PC practices as a consequence of COVID-19 pose a threat to the good organisation of this practice*					
Strongly disagree	11	14.5	413	12.4	0.315
Disagree	32	42.1	1,102	33.0	
Neutral	19	25	898	26.9	
Agree	10	13.1	713	21.3	
Strongly agree	4	5.3	215	6.4	
Total	76		3,341		
Missing	18		2,620		
*The guidelines imposed by the government on PC practices as a consequence of COVID-19 pose a threat to the personal well-being of the staff in this practice*					
Strongly disagree	9	11.4	457	13.7	0.810
Disagree	23	29.1	1,065	31.9	
Neutral	19	24.0	762	22.8	
Agree	22	27.8	758	22.7	
Strongly agree	6	7.6	299	8.9	
Total	79		3,341		
Missing	15		2,620		
*Adequate support is provided by the government for the proper functioning of this practice*					
Strongly disagree	28	34.1	609	18.1	< 0.001
Disagree	29	35.4	1,096	32.6	
Neutral	21	25.6	801	23.8	
Agree	4	4.9	689	20.5	
Strongly agree	0		170	5.1	
Total	82		3,365		
Missing	12				

The Israeli and the entire cohort responses to questions regarding protected time reviewing new data (literature and guidelines), the guidelines imposed by the government and their impact on practices and the support received by the government

**Table 4:** Collateral damage

	Israeli respondents		Entire cohort		*P* value
	n	%	n	%	
*A patient with a fever caused by an infection other than COVID-19 was seen late due to the fact the COVID-19 protocol was followed which delayed the care*					
Yes	55	71.4	1,212	41.4	< 0.001
No	22	28.6	1,718	58.6	
Total	77		2,930		
Missing	17		3,031		
*A patient with an urgent condition was seen late because he/she did not come to the practice sooner*					
Yes	53	76.8	1,768	59.9	0.005
No	16	23.2	1,183	40.1	
Total	69		2,951		
Missing	25		3,010		
*A patient with a serious condition was seen late because he/she did not know how to call on a GP*					
Yes	31	46.3	729	27.2	0.001
No	36	53.7	1,955	72.8	
Total	67		2,684		
Missing	27		3,277		
*A patient with an urgent condition was seen late, because the situation was assessed as non-urgent during the telephone triage*					
Yes	15	27.3	546	20	0.184
No	40	72.7	2,183	80	
Total	55		2,729		
Missing	39		3,232		
*A patient with an urgent condition other than COVID-19 was assessed incorrectly during the triage procedure*					
Yes	17	32.7	763	29.1	0.573
No	35	67.3	1,859	70.9	
Total	52		2,622		
Missing	42		3,339		

The Israeli and the entire cohort responses to questions regarding delays in diagnosis and treatment of non-COVID health problems during the pandemic

**Table 5:** PCPs’ well-being

	Israeli respondents		Entire cohort		*P* value
	n	%	n	%	
*Mayo clinic well-being index – before the pandemic*					
Score < 2 (low risk)	34	60.7	1170	37.8	< 0.001
Score ≥ 2 (increased risk)	22	39.3	1929	62.2	
Total	56		3099		
Missing	38		2862		
*Mayo clinic well-being index – since the pandemic*					
Score < 2 (low risk)	22	41.5	856	29.7	0.062
Score ≥ 2 (increased risk)	31	58.5	2030	70.3	
Total	53		2886		
Missing	41		3075		

The Mayo clinic well-being index is a validated tool for assessing physicians’ well-being as well as identifying an increased risk for burnout. The score ranges from − 2 to 9, with higher scores indicating a higher degree of distress. An “at-risk score” is an index score of two or higher, which indicates higher risk for burnout, severe fatigue, suicidal ideation, and poor overall quality of life

### Telemedicine use (Table 2)

The use of video consultations increased dramatically, 11% of respondents reported that they provided at least one video consultation before the pandemic; this proportion increased to 49% during the pandemic. Most physicians reported that their patients were not instructed regarding which complaints were suitable for telemedicine and which required a face-to-face visit.

### Information and guidelines (Table 3)

#### Secured timeslots

Before the pandemic approximately 3/4 of the respondents indicated that they did not have secured timeslots for reading new guidelines or scientific literature and had no regular staff meetings. During the pandemic, when guidelines were frequently updated, this situation did not change. In the international cohort, a higher proportion of the respondents reported having secured timeslots before and during the pandemic (32% vs. 16%, *p* < 0.001, 32% vs. 18%, *p* < 0.001, respectively).

#### Governmental guidelines

18% of the respondents thought that the guidelines published by the MoH posed a threat to the good organization of their clinics, 35% thought that these guidelines threatened the well-being of the staff in their clinics. This was in line with the results of the international cohort. 69% of the respondents in Israel felt that the government had not provided adequate support for the proper functioning of their clinics, while 51% of respondents of the international cohort felt this way (*p* < 0.001).

### Delays in examination and treatment of non-COVID problems (Table 4)

Due to the complexity and the high degree of uncertainty, patient safety incidents occurred in all practices; more than 70% of the respondents reported that patients with urgent conditions not related to COVID-19 (fever or other conditions) had a delayed examination, 46% of the respondents reported that patients with serious conditions had a delayed examination because they did not know how to contact their PCPs. These incidents were reported more often by PCPs in Israel than in the international cohort. Other incidents, such as patients with urgent conditions that were seen late because they were assessed as non-urgent during a telephonic triage (27%) and patients with urgent conditions other than COVID-19 that were assessed incorrectly during the triage procedure, (33%) were reported in equal proportions in Israel and the international cohort.

### A decline in PCP’s well-being (Table 5)

When asked about their well-being before the pandemic, 39% of the Israeli respondents received an “at-risk score” in the Mayo Clinic Well-being Index score. When asked about their current state (i.e. during the pandemic), this proportion increased to 58%, an increase of 1.49 fold. The correlation of well-being scores before and during the pandemic was high (R = 0.92, *p* < 0.001). A lower proportion of the Israeli respondents had “at-risk scores” than the international respondents, and the difference between the Israeli and the international respondents was higher before the pandemic (39% vs. 62%, *p* < 0.001 respectively) than during the pandemic (58% vs. 70%, *p* = 0.062 respectively). The well-being score during the pandemic correlated with the feeling of being unprepared for the shifting of the physician’s role during the pandemic (Spearman’s r = 0.27, *p* = 0.055), and negatively correlated with feeling that the respondent was happy with their role shifting (Spearman’s r = -0.37, *p* = 0.007).

## Discussion

### Principal findings

We reported the experience of PCPs in Israel during the pandemic and compared it to the experience of PCPs from 29 countries. The main findings of this study were: increased use of telemedicine without sufficient instructions to patients regarding to proper use of this technology; lack of secured time-slots for reviewing new guidelines; and the impact of the MoH’s guidelines on the organization and well-being of clinics and staff. To be noted, many PCPs experienced these guidelines as a threat to their well-being and to the organization of their practices. Other findings of this study include: delayed treatment of non-COVID related conditions and deterioration of physician’s well-being during the pandemic.

### Interpretation

The pandemic resulted in many adaptations. One of the most significant ones was the increase in telemedicine use, especially video consultations [19]. PCPs reported ambivalent feelings regarding the increased use of telemedicine. On one hand, it offers new opportunities to improve patient care. On the other hand, it profoundly changes interactions with patients: physical examination is not possible, communication without physical presence may be harmed and patients may be less willing to disclose their most personal thoughts and concerns, thus affecting their care [20]. A major challenge to future primary care will be to balance between telemedicine and in-person visits; Jabbarpour et.al estimated in their study that about two thirds of all primary care visits required an in-person service [21]. The shift from in-person visits to telemedicine should be addressed by healthcare policy makers and scientific associations in order to provide guidelines regarding which type of visits should be held using which modality. These guidelines should be provided to both PCPs and patients, as this problem was reported by PCPs globally [22–24].

During the pandemic, knowledge regarding the virus and proper treatment for the disease increased rapidly, and although major changes to the daily practice were seen in ambulatory care, not many studies have explored the impact of these changes on physicians who work in the community. In this study, PCPs reported that they did not have secured timeslots for reviewing new guidelines and reported inadequate support from the government for the proper functioning of their clinics. This was especially troublesome during the pandemic when guidelines changed on a weekly and sometimes even daily basis. While some respondents indicated that the guidelines that were introduced by the government posed a threat to the orderly functioning of their clinics and the well-being of the staff, the majority felt that the MoH did not provide adequate support to their clinic during the pandemic. These statements may reflect the distance between the Israeli MoH and community healthcare personnel, which might have originated from lack of proper representation of community healthcare providers at the policymaker level in the ministry of health.

“Collateral damage”, which is the delay in medical treatment due to COVID-19 [25], was reported by many PCPs in our study. They indicated that patients with urgent conditions sometimes failed to seek timely medical advice. Problems were also reported in the triage system, resulting in delayed medical treatment. A possible cause for delayed treatment may be a shift towards automated visit scheduling systems, which in many clinics completely replaced medical secretary staff. In France, most of the patient-safety incidents were related to delay diagnosis, assessment and referral [26]. Other studies have demonstrated that during the pandemic preventive care services, cancer diagnosis and management of cardiovascular diseases were delayed [27–29].

PCP’s well-being during the pandemic deteriorated, as evident from the increase of respondents who were classified as at-risk by the Mayo Clinic Well-Being-Index. The decreased well-being of PCPs that was identified in this study supports the findings of previous studies [30, 31]. This was especially worrisome because in addition to the impact on the physician himself, it also had impact on patient safety, quality of care and satisfaction [32]. Baptista et al. suggested involving physicians in health policy, guidelines and plans as well as providing them with training, a supportive network and emotion management in order to reduce the high burnout rate of PCPs [30].

### Strengths and limitations

The questionnaire was answered from December 2020 to July 2021, thus capturing the physician’s sentiment during the third wave (alpha variant) and the beginning of the fourth wave (delta variant) of the pandemic in Israel. This allowed us to get first-hand real-time reactions of community physicians, who treated the majority of the COVID-19 patients.

The main limitation of the study was its sampling method that was also used by the international study. Using a convenience sample of physicians who were more involved in teaching and in peer consulting provided responses of mainly family medicine specialists, who may not represent all PCPs in Israel. We suspect that the respondents may be more resilient to stress and changes, due to their involvement in teaching in addition to practicing clinical medicine [33]. The conferences took place in the center of Israel and were relatively small, which might also create a bias towards overrepresentation of physicians who live and work in the center of Israel. Some of the questions received lower response rates compared with the rest of the questionnaire, missing answers are reported separately for each of the questions in Table 2, 3, 4 and 5. The Israeli respondents were comprised by 47 (50%) family medicine specialists and 18 trainees (19.1%). The entire population of PCPs in Israel is comprised by only one third of family medicine specialists (with 20% internal medicine specialists and almost half without any specialty) [34].

As the survey enquired about the participants` experience rather than asking directly about their age, we estimated the age from the reported experience. 30% of our respondents reported 0–10 years of experience (estimated age 30–40) compared to less than 15% in the entire population of Israeli PCPs, 15% reported 10–20 years of experience (estimated age 40–50) compared to 22% in the entire population of Israeli PCPs, 10% reported 20–30 years of experience (estimated age 50–60) compared to 30% of the entire population of Israeli PCPs, and 6.5% reported 30–35 years of experience (presumed age 60–65) compared to 27.8% of the entire population of Israeli PCPs [34].

The well-being score before the pandemic was calculated using retrospective question what might reduce its validity.

### Implications for health policymakers

The pandemic had a major impact on health care organizations and on primary care specifically. There is a paucity of research about the impact of the pandemic on PCPs. While the findings of this study raise concerns regarding the PCPs experience during the pandemic, they provide opportunities for adaptation and improved processes of care.

As telemedicine becomes acceptable and common practice, guidelines for proper visit management and substitutes for the physical examination should be developed and taught to physicians. Scheduling systems should provide guidelines and educational information to the patients regarding the preferred form of medical encounter for their current complaint. During times of crisis, governments may also consider communicating this information through public media in order to reduce the collateral damage that originated from delayed diagnosis due to patients delayed seeking medical help. Government directives and clinical guidelines, especially during a pandemic, should be communicated in a timely manner, with either secured timeslots for physicians’ self-learning or regular staff meetings.

Last but not least, as seen in this study, physicians’ well-being deteriorated during the pandemic; ensuring physicians’ well-being in general should be an organizational priority and may prevent its deterioration during a pandemic. Future research should be focused on ways to ensure physicians’ well-being, on how to update physicians in a timely manner, and to clarify the physicians’ perception of government directive as a threat to their practices.

## Conclusion

In this study, we reported the experiences of PCPs in Israel during the COVID-19 pandemic. We raised major concerns regarding PCPs’ preparation, information flow, collateral damage, and deterioration of PCPs’ well-being.

As PCPs are at the forefront of treating patients, special attention should be given to the adaptation of the primary care system, staff preparation, and public education regarding telemedicine; in addition, secured timeslots for PCPs` to update on new guidelines and instructions should be provided. Procedures for minimizing collateral patient damage and reaching out to patients at-risk for domestic violence or psychological distress should be developed and adopted. Physicians` well-being was not properly addressed, and it should become an important consideration for policymakers in order to ensure continuity of primary care in the community.

## Supplementary Information

**Additional file 1. **The Mayo Clinic Well-being Index ©. Description: This is the English version of the Mayo Clinic Well-being Index ©.

**Additional file 2. **The English version of the PRICOV questionnaire. Description: This is the English version of the PRICOV questionnaire.

## Data Availability

The datasets generated and analyzed during the current study are not publicly available due to ethical considerations, but are available from the corresponding author on reasonable request.

## Abbreviations

HMOs: Healthcare maintenance organizations
PCP: Primary care physician
MoH: Ministry of health
