# Supplementary material for: The effect of the COVID-19 pandemic on primary care physicians in Israel, with comparison to an international cohort: a cross-sectional study
Source: Isr J Health Policy Res. 2022 Sep 20;11:34. doi: 10.1186/s13584-022-00543-8 (PMC9486777; doi:10.1186/s13584-022-00543-8)
Supplement: Supplementary file 1 — Additional file 1. The Mayo Clinic Well-being Index ©. Description: This is the English version of the Mayo Clinic Well-being Index ©. [file 13584_2022_543_MOESM1_ESM.docx]

**Additional File 1 -** © Mayo Clinic Well-being Index

During the past month...

1. Have you felt burned out from your work? Yes / no

2. Have you worried that your work is hardening you emotionally? Yes / no

3. Have you often been bothered by feeling down, depressed, or hopeless? Yes / no

4. Have you fallen asleep while sitting inactive in a public place? Yes / no

5. Have you felt that all the things you had to do were piling up so high that you could not overcome them? Yes / no

6. Have you been bothered by emotional problems (such as feeling anxious, depressed, or irritable)? Yes / no

7. Has your physical health interfered with your ability to do your daily work at home and/or away from home? Yes / no

8. Please rate how much you agree with the following statements:

1 (Stongly disagree) 2 3 4 5 6 7 (Strongly agree)

1. The work I do is meaningful to me.

2. Since the COVID-19 pandemic, the work I do has become more meaningful to me.

9. Please rate how much you agree with the following statements:

1 (Stongly disagree) 2 3 4 5 (Strongly agree)

1. My work schedule leaves me enough time for my personal/ family life.
2. Since the COVID-19 pandemic, my work leaves me enough time for my personal/ family live.

Permission to use this questionnaire was granted by the author of the questionnaire to the lead researchers in Ghent University.
